# Excessive Smartphone Use as a Risk Factor for Clinical Signs Compatible with De Quervain’s Tenosynovitis: A Cross-Sectional Study

**DOI:** 10.1007/s43465-025-01629-6

**Published:** 2025-11-27

**Authors:** Bartosz Pomianowski, Leon Smółka, Karolina Blady, Miłosz Strugała, Karolina Kursa, Agnieszka Woźniak

**Affiliations:** 1 Department of Orthopedics and Traumatology, Medical University of Silesia, Katowice, Poland; 2 Department of Human Anatomy, Faculty of Medical Sciences in Katowice, Medical University of Silesia, Katowice, Poland

**Keywords:** De Quervain tenosynovitis, Smartphone overuse, Young population, Musculoskeletal strain

## Abstract

**Background:**

Excessive smartphone use has become an integral part of daily life among young adults, potentially leading to repetitive strain conditions involving the tendons of the wrist. One of the most frequent disorders linked to such overuse is de Quervain’s tenosynovitis, which affects the tendons within the first dorsal compartment. As mobile device use continues to rise worldwide, understanding its musculoskeletal implications is essential for prevention and early clinical management.

**Aim:**

This study aimed to examine the association between smartphone usage patterns—specifically screen time and unlocking frequency—and clinical signs consistent with de Quervain’s tenosynovitis in young adults, with special attention to the dominant hand.

**Materials and Methods:**

Data were obtained from an original cross-sectional survey assessing smartphone usage behaviors, addiction severity (Smartphone Addiction Scale-Short Version, SAS-SV), and musculoskeletal symptoms using the Finkelstein maneuver. A total of 202 participants were included in the analysis. Statistical evaluation employed Spearman’s rank correlation, Mann–Whitney U tests, and logistic regression to identify relationships between device usage, addiction level, and pain occurrence in the dominant hand.

**Results:**

The findings demonstrated a significant positive association between the intensity of smartphone use and self-reported wrist pain. Pain correlated moderately with both the number of phone unlocks (*ρ* = 0.596, *p* < 0.001) and daily screen time (*ρ* = 0.524, *p* < 0.001). Participants classified as addicted according to the SAS-SV scale exhibited greater usage intensity, which corresponded with increased pain prevalence.

**Conclusions:**

Frequent and prolonged smartphone use, reflected by higher unlocking frequency and screen exposure time, was significantly associated with clinical features suggestive of de Quervain’s tenosynovitis in the dominant hand. Users meeting the criteria for smartphone addiction showed greater symptom severity. The diagnosis was based exclusively on clinical examination without imaging confirmation.

## Introduction

The first smartphones were introduced in 2007 [1]. Over the past two decades, both the number of individuals owning smartphones and the amount of time spent using them have significantly increased [2]. A study conducted by the National Media Institute revealed that in the 16–29 age group, as many as 96.42% of Poles use a smartphone [3]. In addition to offering a wide range of useful features, such as unlimited access to information sources and GPS navigation [4], smartphones also have an impact on human health. They may lead to behavioral addictions, which can manifest as compulsive shopping or gambling [5]. Moreover, smartphones can significantly affect the musculoskeletal system, increasing the risk of developing de Quervain’s disease (DD) [6]. Recent studies have demonstrated that prolonged smartphone use exerts adverse effects on the musculoskeletal system. One of the most commonly reported conditions is neck pain (“text neck”), which arises from a forward head posture, thereby increasing cervical muscle load and resulting in fatigue and discomfort. Moreover, a higher frequency of text messaging has been associated with an elevated risk of neck pain and constitutes an unfavorable prognostic factor with regard to therapeutic outcomes [7]. At the same time, excessive smartphone use may lead to overloading of the thumb muscles, particularly within the thenar region. Intensive use of touch functions, such as screen scrolling and text messaging, can result in microtrauma and inflammatory changes of the tendons, thereby increasing the risk of developing de Quervain’s disease [8]. This condition is primarily caused by excessive and repetitive thumb movements. During simultaneous dorsal flexion and radial deviation of the wrist, friction occurs between the tendons of the abductor pollicis longus (APL) and the extensor pollicis brevis (EPB) muscles and the walls of their shared sheath—the first dorsal compartment. This friction leads to microtrauma, inflammation of the tendon sheaths, and narrowing of the compartment, which predisposes the structures to further damage and intensification of symptoms [9, 10]. The Finkelstein test is widely used in clinical practice to assess clinical signs suggestive of DD [11]. The Finkelstein test is performed by stabilizing the patient’s thumb with one hand while the examiner uses the other hand to gently pull the wrist in the ulnar direction, along the axis of the forearm. If pain occurs on the radial side of the wrist during this maneuver, it may indicate tenosynovitis within the first extensor compartment (de Quervain’s disease) [12]. The test demonstrates higher specificity compared to the Eichhoff test. Studies have shown that the specificity of the Finkelstein test is 100%, while the Eichhoff test reaches 89% [11, 13]. Although the Finkelstein test demonstrates high specificity, it is a clinical maneuver and does not provide definitive diagnosis. Instrumental confirmation, such as ultrasound or MRI, would be required for a conclusive diagnosis of de Quervain’s tenosynovitis. Despite the increasing prevalence of DD among young adults, there remains a notable lack of studies examining the relationship between smartphone usage time, addiction levels, and the number of phone unlocks—each of which involves wrist dorsiflexion and ulnar deviation. Epidemiological studies report that the prevalence of de Quervain’s tenosynovitis in the general population is approximately 0.5% in men and up to 1.3% in women, depending on the study population and the diagnostic criteria used. In certain occupational groups, such as physiotherapists or athletes, the prevalence may be considerably higher, reaching over 40% in some studies [14].

This low baseline prevalence highlights the need for studies investigating early clinical signs in high-risk groups, such as frequent smartphone users. In this study, we focused on young adults, as they represent the group with the highest intensity of smartphone use and most frequently perform repetitive thumb and wrist movements. This exposure places them at increased risk of developing de Quervain’s disease. Furthermore, in Poland, the proportion of smartphone users within this age group is among the highest in the population, making it a particularly relevant target for investigation.

We hypothesized that longer daily smartphone use, higher levels of smartphone addiction, and a greater number of phone unlocks are independently associated with an increased prevalence of de Quervain’s disease among young adults.

## Methodology

A cross-sectional survey was conducted among young adults aged 29 years or younger in Poland. Specifically, participants were recruited within the Silesian metropolitan area using a convenience sampling method across various settings, including university campuses, hospital classes, social venues, and public spaces. The study was conducted between March 4 and April 9, 2025. Participants were directly approached in the aforementioned settings and invited to take part if they met the inclusion criteria. Recruitment was voluntary and conducted through face-to-face contact during academic activities, hospital classes, social gatherings, and in public areas. The aim of this study was to investigate the correlation between smartphone use and clinical signs suggestive of de Quervain’s tenosynovitis in young adults. The primary objective was to assess the relationship between objective smartphone use metrics (average daily screen time and number of unlocks per day) and the presence of clinical signs suggestive of de Quervain’s tenosynovitis, as indicated by a positive Finkelstein test. Participation was anonymous, voluntary, and preceded by informed consent.

Data collection was carried out in two stages:

1. Online survey

Participants completed a custom-designed online questionnaire, which included questions about:

- demographic data (age, gender),
- average daily smartphone use (based on device-provided data),
- average number of daily screen unlocks (also based on device-provided data),
- the preferred hand and finger used to operate and unlock the smartphone,
- the level of smartphone addiction assessed using the Smartphone Addiction Scale-Short Version (SAS-SV)*,
- any history of wrist injuries or diagnosed degenerative conditions.

*Smartphone addiction was assessed using the official English version of the Smartphone Addiction Scale-Short Version (SAS-SV). All participants demonstrated sufficient English language proficiency to provide reliable responses. For individuals less confident in English, a Polish translation prepared by the research team was available as supportive material; however, no participant required its use.

To ensure data accuracy, information on daily screen time and number of unlocks was obtained directly from the built-in smartphone usage statistics. Participants were asked to show these statistics to the investigator at the time of survey completion, which minimized the risk of recall bias or subjective misreporting. Smartphone screen time and number of daily unlocks were analyzed primarily as continuous variables. For descriptive purposes and subgroup comparisons, these variables were additionally categorized into quartiles. Smartphone addiction was classified according to validated SAS-SV cut-offs (≥33 points for females, ≥31 for males).

2. Clinical assessment—Finkelstein’s test

Each participant underwent the Finkelstein test, a standard diagnostic maneuver used to assess de Quervain’s tenosynovitis. It was performed by making a fist over the patient’s thumb and then gently pulling the wrist in the ulnar direction, along the axis of the forearm, which passively stretched the tendons. The test evaluates tenderness over the tendons of the extensor pollicis brevis and abductor pollicis longus muscles.

To ensure maximum reliability and consistency, all assessments were performed by the same examiner following a standardized protocol. The Finkelstein’s test was performed by an orthopedic surgeon with 3 years of professional experience. Previous studies have demonstrated that the Finkelstein test has high diagnostic accuracy in detecting de Quervain’s tenosynovitis, with reported specificity approaching 100% and higher reliability compared to the Eichhoff test. Therefore, its application in this study provided a valid and reliable clinical measure for identifying symptomatic individuals.

Results were classified as follows:

- Bilateral negative—no pain symptoms on either side.
- Unilateral positive—pain present on one side only (interpreted as relevant if it occurred on the hand used to unlock the smartphone), meaning discomfort was noted in one hand but absent in the other.
- Bilateral positive—significant pain and overload symptoms on both sides.

3. Inclusion and exclusion criteria

Only participants meeting all the following criteria were included in the final analysis:

- Adults aged under 30 years.
- No reported wrist injuries or degenerative wrist conditions.
- Ownership of a smartphone that provides data on screen time and number of unlocks.
- Preferential use of the thumb for both operating and unlocking the smartphone.

Participants were excluded if they:

- Exceeded the age limit.
- Reported wrist injury or degenerative disease.
- Used a finger other than the thumb (most often the index finger).
- Were unable to retrieve usage data from their device (e.g., due to lack of tracking features).

It should be noted that other potential confounding factors, such as participation in sports involving repetitive wrist and thumb movements (e.g., badminton, tennis) or the presence of chronic musculoskeletal conditions not reported by participants, were not systematically assessed and may have influenced the outcomes.

Assessment of clinical signs suggestive of de Quervain’s tenosynovitis based exclusively on clinical testing has several important limitations. Pain response during the test is subjective, so it might be hard to assess as the patient reporting is influenced by the individual pain threshold. Pain may also be elicited in other conditions affecting the wrist or the thumb. Even though participants were excluded if they reported previous wrist injuries or degenerative diseases affecting the wrist, there might be some conditions, such as ganglion cysts, which may sometimes remain undiagnosed and can mimic similar pain patterns during the Finkelstein’s test.

A post hoc power analysis was conducted to assess the adequacy of the final sample. With *N* = 155 participants, the study had over 99% power to detect the observed correlations between smartphone use and positive Finkelstein test results (*r* = 0.52–0.60) at a two-tailed significance level of *α* = 0.05. The minimal detectable effect size at 80% power was approximately |*r*|= 0.22, indicating that the study was sufficiently powered to detect moderate associations between smartphone use and clinical signs suggestive of de Quervain’s tenosynovitis.

The findings were discussed in relation to the existing literature on the association between smartphone use and wrist pain, a symptom characteristic of de Quervain’s tenosynovitis. Data were analyzed using Python 3.13 with the following libraries: pandas, numpy, seaborn, matplotlib, scipy.stats, and statsmodels. All correlation coefficients are reported with 95% confidence intervals (CI) calculated using Fisher’s z-transformation.

Nonparametric tests (Spearman’s rank correlation, Mann–Whitney U test, *χ*^2^ test, and two-proportion *Z* test) were applied due to non-normal distributions of smartphone use variables. To account for the risk of type I error in multiple comparisons, a Bonferroni correction was applied in exploratory analyses. Assumptions of the statistical tests were verified by visual inspection of variable distributions. Sensitivity analyses were conducted by excluding participants with extreme smartphone usage values (>3 standard deviations from the mean) to assess the robustness of the results.

## Results

**Fig. 1:**
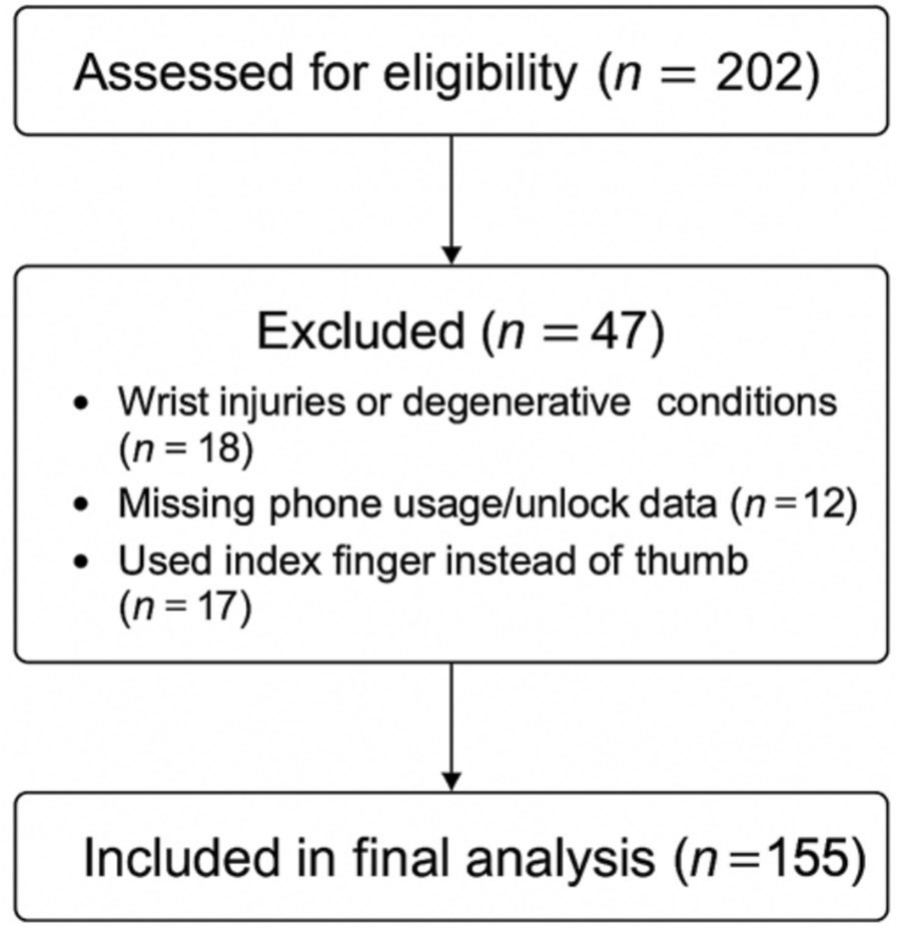
Flowchart of participant recruitment

A total of 202 individuals participated in the study by completing the survey and providing full responses. Subsequently, data were filtered according to the predefined methodology (Fig. 1).

The final sample comprised 155 young adults aged 30 years or younger (mean age: 21.5 ± 1.84 years). The majority of participants were female (65.2%), and right-hand dominance was reported by 93.5% of the respondents. On average, participants reported using their smartphones for 4.9 ± 1.7 h per day and unlocking the screen approximately 101 ± 65 times per day (median: 125 unlocks). Based on the Smartphone Addiction Scale-Short Version (SAS-SV), 40 individuals (25.8%) met the established criteria for smartphone addiction (≥33 points for females, ≥31 for males).

A positive Finkelstein test, indicating clinical signs suggestive of de Quervain’s tenosynovitis, was observed in 51 participants (32.9%). A comparison between included participants (*n* = 155) and those excluded due to predefined criteria (*n* = 47) showed no significant differences in age or gender distribution (*p* > 0.05). This suggests that the final analytical sample was broadly representative of the recruited group. However, since participants were primarily volunteers, the possibility of selection bias cannot be fully excluded.

Detailed demographic and clinical characteristics of the sample are presented in Table 1.

**Table 1:** Demographic and clinical characteristics of the study participants

Variable	Description *n* (%)
Sex	Female 101 (65.2%) Male 54 (34.8%)
Smartphone addiction	Addicted 40 (25.8%) Not addicted 115 (74.2%)
Positive Finkelstein test (clinical signs suggestive of de Quervain’s tenosynovitis—unlocking hand)	Positive 51 (32.9%) Negative 104 (67.1%)
Dominant hand	Right 145 (93.5%) Left 10 (6.5%)

Spearman’s rank correlation coefficients demonstrated significant positive associations between the extent of smartphone use and the severity of addiction symptoms:

- Screen time vs. SAS-SV: *ρ* = 0.210 (95% CI 0.05–0.36), *p* = 0.0044.
- Unlock frequency vs. SAS-SV: *ρ* = 0.235 (95% CI 0.08–0.38), *p* = 0.0023.

Stronger correlations were observed between smartphone use and clinical signs suggestive of de Quervain’s tenosynovitis (positive Finkelstein test results):

- Screen time vs. positive Finkelstein test: *ρ* = 0.524 (95% CI 0.40–0.63), *p* < 0.0001.
- Unlock frequency vs. positive Finkelstein test: *ρ* = 0.596 (95% CI 0.48–0.69), *p* < 0.0001.

Nonparametric Mann–Whitney U tests confirmed significantly higher screen time (*U* = 6442.0, *p* < 0.0001) and number of daily unlocks (*U* = 5456.5, *p* < 0.0001) among participants with a positive Finkelstein test. Although these results were statistically significant, the effect sizes indicate clinically meaningful differences. For instance, the correlation between unlock frequency and positive Finkelstein test was large (*ρ* = 0.596), while that between screen time and Finkelstein test was moderate to large (*ρ* = 0.524). Clinically, this suggests that individuals exceeding approximately 200 unlocks per day are at substantially higher risk of reporting pain in the dominant hand. These thresholds may therefore serve as useful markers for preventive strategies, complementing purely statistical significance.

Among individuals classified as addicted vs. non-addicted, the difference in screen time approached statistical significance (*U* = 3745.0, *p* = 0.0735), while the number of unlocks per day was significantly higher in the addicted group (*U* = 3212.0, *p* = 0.0234).

There was no statistically significant association between smartphone addiction status and clinical signs suggestive of de Quervain’s tenosynovitis (Table 2) (positive Finkelstein test):

- Chi-squared test: *χ*^2^ = 2.57, *p* = 0.1092.
- Two-proportion *Z*-test: *Z* = -1.77, *p* = 0.0764.

**Table 2:** Summary of statistical comparisons between smartphone use, addiction symptoms, and Finkelstein test (clinical signs suggestive of de Quervain’s tenosynovitis) results

Analysis	Variable 1	Variable 2	Statistic	Value	*p*-value
Spearman correlation	Screen time (h)	Positive Finkelstein test (clinical signs suggestive of de Quervain’s tenosynovitis—unlocking hand)	*ρ*	0.524	<0.0001
Spearman correlation	Unlocks/day	Positive Finkelstein test (clinical signs suggestive of de Quervain’s tenosynovitis—unlocking hand)	*ρ*	0.596	<0.0001
Spearman correlation	Screen time (h)	SAS-SV score	*ρ*	0.210	0.0044
Spearman correlation	Unlocks/day	SAS-SV score	*ρ*	0.235	0.0023
Mann–Whitney U test	Screen time (h)	Smartphone addiction (SAS-SV)	*U*	3745.0	0.0735
Mann–Whitney U test	Unlocks/day	Smartphone addiction (SAS-SV)	*U*	3212.0	0.0234
Mann–Whitney U test	Screen time (hrs)	Positive Finkelstein test (clinical signs suggestive of de Quervain’s tenosynovitis—unlocking hand)	*U*	6442.0	<0.0001
Mann–Whitney U test	Unlocks/day	Positive Finkelstein test (clinical signs suggestive of de Quervain’s tenosynovitis – unlocking hand)	*U*	5456.5	<0.0001
Chi-squared test	Smartphone addiction	Positive Finkelstein test (clinical signs suggestive of de Quervain’s tenosynovitis—unlocking hand)	*χ*^2^	2.57	0.1092
Proportion Z-test	Smartphone addiction	Positive Finkelstein test (clinical signs suggestive of de Quervain’s tenosynovitis—unlocking hand)	*Z*	−1.77	0.0764

Additionally, subgroup analyses revealed that women reported slightly higher average screen time and unlock frequency compared with men, although the differences did not reach statistical significance (*p* > 0.05). Similarly, addicted participants (as defined by the SAS-SV cutoff) demonstrated higher device use and more frequent positive Finkelstein test results than non-addicted participants, consistent with the overall findings. Sensitivity analyses excluding participants with extreme smartphone usage values (>3 SD above the mean) did not materially alter the results, confirming the robustness of the associations. Potential confounders, including sex and hand dominance, were examined but showed no significant interaction with the main outcomes.

## Discussion

Nearly 92% of participants reported using their thumbs to operate and unlock their smartphones. This suggests that, for most users, excessive use of mobile devices may directly contribute to an increased risk of developing inflammatory changes in the tendons of the first extensor compartment. Our results are consistent with previous studies, suggesting a higher occurrence of pain during Finkelstein’s test among women and extensive smartphone users [6, 12]. Moreover, our participants were more likely to be classified as extensive smartphone users according to the SAS-SV than participants in other analyzed populations. It may suggest that young, European students tend to spend more time daily using their phones than individuals from other populations. As reported in the literature, women are more predisposed to developing de Quervain’s tenosynovitis [12], and the prevalence among women is up to three times higher than in men [15]. De Quervain’s disease occurs particularly often in women during the perinatal period, both due to the influence of hormonal changes taking place in the female body and because of the need to carry the baby, which requires frequent, repetitive movements that strain the first dorsal compartment of the wrist extensors [12]. Women are also more likely to work in occupations that demand repetitive thumb movements, which may further exacerbate inflammation in this region. Participants reported using their smartphones for an average of 4.9 h per day, which is slightly lower than the average found in other studies for this age group, where usage exceeded 6 h per day [16]. However, the average number of screen unlocks per day was 124, which is higher than reported in previous research [17], indicating more frequent, though potentially shorter, interactions with the device. The SAS-SV questionnaire used in this study enabled the assessment of smartphone addiction. According to the established thresholds, 25.3% of respondents met the criteria for addiction, with a mean SAS-SV score of 27.9. In comparison, studies in other populations reported lower scores—for example, an average of 25.75 in a Japanese adult population [18] and 22.28 among Hungarian university students, who had a similar average age (21.9 vs. 21.5 years) [19]. The percentage of addicted individuals in both of these studies was also lower, at 14.9%. A similar addiction rate of 14.9% was found among high school students in Tunisia [20]. In a study by Harris et al. [21] conducted among Texas university students, the mean SAS-SV score was 24.53—again, lower than in our sample. These comparisons suggest that the proportion of addicted individuals in our study was higher than in both European and non-European samples. Furthermore, a statistically significant, moderately positive correlation (*ρ* = 0.210; *p* < 0.005) was observed between SAS-SV score and daily smartphone use. This indicates that psychological dependence tends to develop more frequently in individuals who overuse their phones—an observation supported by other studies [22, 23]. Nikolic et al. [24] also reported a similar statistically significant, moderate positive correlation between screen time and SAS-SV scores, supporting the validity of the scale as a diagnostic tool for smartphone addiction. Our results indicate that pain during the Finkelstein test occurred more frequently in individuals who used their smartphones for longer periods and more often in the dominant (unlocking) hand. This is likely due to increased tension in the first extensor compartment caused by repetitive thumb movements. Pain was also more common among those who unlocked their phones more frequently. The growing prevalence of smartphone overuse has been associated with increased reports of thumb pain and reduced mobility, especially in the dominant hand, as noted in previous literature [25, 26]. We also observed that pain in the dominant hand was associated with both longer usage time and higher unlock frequency. In fact, high unlock frequency combined with prolonged daily use was almost always accompanied by pain during testing. Among individuals who unlocked their phones more than 200 times per day, pain was common even when overall screen time was lower. Finkelstein test results demonstrated a positive correlation between unlock frequency and reported pain in the dominant hand. Participants who unlocked their phones less frequently rarely experienced positive test results, even if they used their phones for more than 5 h daily. In our study a dose–response relationship was noticed, due to the fact that participants who spent more time using their phones and unlocked them more frequently were more likely to report the pain during Finkelstein’s test. It suggests that people who can be classified as extensive smartphone users should be observed due to the higher risk of developing de Quervain’s disease in the future. The frequency of de Quervain’s disease appears to be markedly higher among individuals who are heavy smartphone users. This correlation has been reported by Rehan Asad et al. [6], who found that frequent texting was associated with a higher prevalence of wrist pain. Similarly, Xinyu Nie et al. [27] observed that the risk of developing de Quervain’s tenosynovitis was highest among students with the longest screen time. That study also highlighted differences in smartphone usage purposes. Among heavy users, a significant portion of screen time was spent playing mobile games—a behavior that may further promote repetitive thumb movements and increase the risk of overuse injuries. Our study shows that Finkelstein’s test can be a useful tool and a part of preventive strategy for developing DD in the future, as it may be used for early identification for individuals who are at high risk of the disease. Even though the Finkelstein’s test remains a valuable and useful screening tool, future studies should include instrumental diagnostic confirmation of symptoms compatible with de Quervain’s disease, as both MRI and ultrasound will help to provide detailed information about inflammatory changes in the first compartment of the wrist extensors, may visualize tendon sheath thickening and can distinguish the disease from other conditions affecting the wrist.

## Limitations

This study has several limitations. First, its cross-sectional design precludes any inference about causality, as only associations could be established. Second, the assessment relied exclusively on the Finkelstein test without instrumental confirmation (e.g., ultrasound or MRI). Although this clinical test has high specificity, it may yield false-positive results and cannot distinguish de Quervain’s tenosynovitis from other tendinopathies or painful wrist conditions. Third, symptom severity was not formally assessed, which limits the interpretation of clinical burden. Fourth, *selection bias* may have influenced the results, as participants were mainly recruited from university populations and may not be representative of all young adults. In addition, *information* and *recall bias* are possible due to the self-reported nature of certain variables (e.g., hand use, injury history). Although strict inclusion and exclusion criteria were applied, objective device-generated usage data were used, and all clinical examinations were standardized and performed by a single trained examiner, the possibility of residual confounding cannot be excluded. Finally, the findings may have limited generalizability beyond young adults in Poland. Future studies should include more diverse populations, assess symptom severity, and incorporate imaging confirmation to establish definitive diagnostic criteria and to inform evidence-based diagnostic protocols.

## Summary

In summary, our study highlights an increased risk of developing de Quervain’s disease among individuals who use smartphones intensively. The risk of pain in the first extensor compartment appears to rise with both longer usage time and higher frequency of phone unlocks; the risk applies to the population using the thumb to unlock and operate the phone. The long-term impact of smartphone use on the incidence of de Quervain’s tenosynovitis should be investigated using more precise diagnostic tools and a broader set of variables. It is essential to more accurately profile the time spent on specific activities using mobile devices, including detailed monitoring of the number of characters typed in messaging apps, web browsers, and other applications that require intensive thumb use. Such analyses should be complemented by a comprehensive assessment of participants’ daily functioning profiles—including physical activity, nature of work, daily habits, and other environmental and behavioral factors that may contribute to the clinical signs suggestive of de Quervain’s tenosynovitis. Proper identification and reduction of risk factors could serve as an effective prevention strategy, which—given the widespread and growing use of smartphones—may lead to meaningful and measurable public health benefits. Further studies with instrumental confirmation are needed to establish definitive diagnostic criteria. 

## Data Availability

The datasets generated and analyzed during the current study are not publicly available due to privacy and ethical restrictions but are available from the corresponding author on reasonable request.

## References

[1] Wajcman, J. (2008). Life in the fast lane? Towards a sociology of technology and time. *British Journal of Sociology,**59*(1), 59–77. 10.1111/j.1468-4446.2007.00182.x 18321331 10.1111/j.1468-4446.2007.00182.x

[2] Ratan, Z. A., Parrish, A. M., Zaman, S. B., Alotaibi, M. S., & Hosseinzadeh, H. (2021). Smartphone addiction and associated health outcomes in adult populations: A systematic review. *International Journal of Environmental Research and Public Health,**18*(22), Article 12257. 10.3390/ijerph182212257 34832011 10.3390/ijerph182212257 PMC8622754

[3] Krajowa Rada Radiofonii i Telewizji. (2024). 75,8 proc. Polaków korzysta ze smartfona, z tabletu – 12,3 proc. [Internet]. gov.pl; 2024 Apr 18 [cited 2025 May 10]. Available from: https://www.gov.pl/web/krrit/758-proc-polakow-korzysta-ze-smartfona-z-tabletu-123-proc

[4] Choi, J. S., Yi, B., Park, J. H., Choi, K., Jung, J., Park, S. W., & Rhee, P. L. (2011). The uses of the smartphone for doctors: An empirical study from Samsung medical center. *Healthcare Informatics Research,**17*(2), 131–138. 10.4258/hir.2011.17.2.131 21886874 10.4258/hir.2011.17.2.131 PMC3155170

[5] Grant, J. E., Schreiber, L. R., & Odlaug, B. L. (2013). Phenomenology and treatment of behavioural addictions. *Canadian Journal of Psychiatry,**58*(5), 252–259. 10.1177/070674371305800502 23756285 10.1177/070674371305800502

[6] Asad, M. R., Ahmad, R. K., Almalki, H. A., Alkhathami, K. M., & Alqahtani, B. (2024). Prevalence of de Quervain’s tenosynovitis among teenage mobile users: A cross-sectional study. *Journal of Pharmacy & Bioallied Sciences,**16*(Suppl 4), S3341–S3344. 10.4103/jpbs.jpbs_823_24 39926998 10.4103/jpbs.jpbs_823_24 PMC11805078

[7] Xie, Y., Szeto, G. P. Y., Dai, J., & Madeleine, P. (2016). A comparison of muscle activity in using touchscreen smartphone among young people with and without chronic neck–shoulder pain. *Ergonomics,**59*, 61–72. 10.1080/00140139.2015.1056237 26218600 10.1080/00140139.2015.1056237

[8] de Jesus Correia, F., Soares, J. B., dos Anjos Matos, R., Pithon, K. R., Ferreira, L. N., & de Assunção, P. L. (2023). Smartphone addiction, musculoskeletal pain and functionality in university students – a observational study. *Psychology, Health & Medicine,**29*, 286–296. 10.1080/13548506.2023.2176893 10.1080/13548506.2023.2176893 36803275

[9] Piver, J. D., & Raney, R. B. (1952). De Quervain’s tendovaginitis. *American Journal of Surgery,**83*(5), 691–694. 10.1016/0002-9610(52)90304-8 14914998 10.1016/0002-9610(52)90304-8

[10] Keon-Cohen, B. (1951). De Quervain’s disease. *Journal of Bone and Joint Surgery. British Volume,**33*, 96–99. 10.1302/0301-620X.33B1.96 14814168 10.1302/0301-620X.33B1.96

[11] Goubau, J. F., Goubau, L., Van Tongel, A., Van Hoonacker, P., Kerckhove, D., & Berghs, B. (2014). The wrist hyperflexion and abduction of the thumb (WHAT) test: A more specific and sensitive test to diagnose de Quervain tenosynovitis than the Eichhoff’s Test. *Journal of Hand Surgery. European Volume,**39*(3), 286–292. 10.1177/1753193412475043 23340762 10.1177/1753193412475043

[12] Fakoya, A. O., Tarzian, M., Sabater, E. L., Burgos, D. M., & Maldonado Marty, G. I. (2023). De Quervain’s disease: A discourse on etiology, diagnosis, and treatment. *Cureus,**15*(4), Article e38079. 10.7759/cureus.38079 37252462 10.7759/cureus.38079 PMC10208847

[13] Wu, F., Rajpura, A., & Sandher, D. (2018). Finkelstein’s test is superior to Eichhoff’s test in the investigation of de Quervain’s disease. *Journal of Hand and Microsurgery,**10*(2), 116–118. 10.1055/s-0038-1626690 30154628 10.1055/s-0038-1626690 PMC6103758

[14] Wolf, J. M., Sturdivant, R. X., & Owens, B. D. (2009). Incidence of de Quervain’s tenosynovitis in a young, active population. *Journal of Hand Surgery. American Volume,**34*, 112–115. 10.1016/j.jhsa.2008.08.020 10.1016/j.jhsa.2008.08.020 19081683

[15] Satteson, E., & Tannan, S.C. (2023). De Quervain Tenosynovitis. In: StatPearls [Internet]. Treasure Island (FL): StatPearls Publishing; 2023 [cited 2025 May 10]. Available from: https://www.ncbi.nlm.nih.gov/books/NBK442005/

[16] Daniyal, M., Javaid, S. F., Hassan, A., & Khan, M. A. B. (2022). The relationship between cellphone usage on the physical and mental wellbeing of university students: A cross-sectional study. *International Journal of Environmental Research and Public Health,**19*(15), 9352. 10.3390/ijerph19159352 35954709 10.3390/ijerph19159352 PMC9368281

[17] Rodman, A. M., Burns, J. A., Cotter, G. K., Ohashi, Y. B., Rich, R. K., & McLaughlin, K. A. (2024). Within-person fluctuations in objective smartphone use and emotional processes during adolescence: An intensive longitudinal study. *Affective Science,**5*(4), 332–345. 10.1007/s42761-024-00247-z 39649461 10.1007/s42761-024-00247-z PMC11624186

[18] Hamamura, T., Kobayashi, N., Oka, T., Kawashima, I., Sakai, Y., Tanaka, S. C., & Honjo, M. (2023). Validity, reliability, and correlates of the Smartphone Addiction Scale-Short Version among Japanese adults. *BMC Psychology,**11*(1), 78. 10.1186/s40359-023-01095-5 36959621 10.1186/s40359-023-01095-5 PMC10034913

[19] Tóth, B., Makai, A., Gyuró, M., Komáromy, M., & Császár, G. (2024). The validity and reliability of the Hungarian version of smartphone addiction scale – Short version (SAS-SV-HU) among university students. *Computers in Human Behavior Reports*, *16*. 10.1016/j.chbr.2024.100527

[20] Yaakoubi, M., Farhat, F., Bouchiba, M., Masmoudi, L., Trabelsi, O., Ghorbel, A., & Gharbi, A. (2024). Smartphone addiction is associated with poor sleep quality, increased fatigue, impaired cognitive functioning, and lower academic achievement: Data from Tunisian middle school students. *School Mental Health,**16*, 1236–1246. 10.1007/s12310-024-09689-z

[21] Harris, B., McCredie, M., & Fields, S. (2020). Examining the psychometric properties of the Smartphone Addiction Scale and its short version for use with emerging adults in the U.S. *Computers in Human Behavior,**1*, Article 100011. 10.1016/j.chbr.2020.100011

[22] Bhalerao, M. M., Krishnan, B., Mokal, S. J., & Latti, R. G. (2020). An analysis of smartphone addiction among MBBS students. *Indian Journal of Clinical Anatomy and Physiology,**7*, 1–7. 10.18231/j.ijcap.2020.001

[23] Servidio, R., Griffiths, M. D., Di Nuovo, S., Sinatra, M., & Monacis, L. (2023). Further exploration of the psychometric properties of the revised version of the Italian smartphone addiction scale – short version (SAS-SV). *Current Psychology,**42*, 27245–27258. 10.1007/s12144-022-03852-y

[24] Nikolic, A., Bukurov, B., Kocic, I., Soldatovic, I., Mihajlovic, S., Nesic, D., Vukovic, M., Ladjevic, N., & Grujicic, S. S. (2022). The validity and reliability of the Serbian version of the Smartphone Addiction Scale-Short Version. *International Journal of Environmental Research and Public Health,**19*(3), 1245. 10.3390/ijerph19031245 35162268 10.3390/ijerph19031245 PMC8835088

[25] Ma, T., Song, L., Ning, S., Wang, H., Zhang, G., & Wu, Z. (2019). Relationship between the incidence of de Quervain’s disease among teenagers and mobile gaming. *International Orthopaedics,**43*(11), 2587–2592. 10.1007/s00264-019-04389-9 31463625 10.1007/s00264-019-04389-9

[26] Baabdullah, A., Bokhary, D., Kabli, Y., Saggaf, O., Daiwali, M., & Hamdi, A. (2020). The association between smartphone addiction and thumb/wrist pain: A cross-sectional study. *Medicine,**99*(10), Article e19124. 10.1097/MD.0000000000019124 32150053 10.1097/MD.0000000000019124 PMC7478614

[27] Nie, X., Huang, L., Hou, J., Dai, A., He, L., Zheng, P., Ye, Z., Zhang, S., Zhou, G., Zhang, J., & Hua, Q. (2023). Smartphone usage behaviors and their association with De Quervain’s Tenosynovitis (DQT) among college students: A cross-sectional study in Guangxi, China. *BMC Public Health,**23*(1), Article 2257. 10.1186/s12889-023-16808-z 37974168 10.1186/s12889-023-16808-z PMC10652590

